# Platinum and Palladium Accumulation in Edible Mushroom *Boletus aereus* Bull. Growing in Unpolluted Soils of Sicily Region (Italy)

**DOI:** 10.3390/jof9090914

**Published:** 2023-09-09

**Authors:** Maria Grazia Alaimo, Daniela Varrica

**Affiliations:** Dipartimento Scienze della Terra e del Mare (DiSTeM), Via Archirafi 22, 90123 Palermo, Italy; daniela.varrica@unipa.it

**Keywords:** PGE, edible mushrooms, bioconcentration factor, daily intake rate

## Abstract

Human exposure to certain metals occurs indirectly through diet. This study was conducted to determine the content of Pt and Pd in fruiting bodies of *Boletus aereus* Bull. collected from several wooded areas of Sicily with different substrates (sedimentary and volcanic) with limited anthropogenic influence. Determinations were performed by coupled plasma-mass spectrometry (ICP–MS) to quantify Pt and Pd. The concentrations of investigated Pt and Pd in mushroom samples ranged from 0.31 to 3.09 ng g^−1^ for palladium and 0.21 to 4.22 ng g^−1^ for platinum. The results of the present study suggest that commonly consumed *Boletus aereus* mushrooms do not accumulate significant levels of Pt and Pd as demonstrated by bioconcentration factor (BCF) values, and their content is lower than in other food products. Additionally, based on the calculated daily intake rates of Pt and Pd, it can be concluded that occasional consumption of fruiting bodies of *B. aereus* collected in Sicily is safe. The proposed methodological approach appears to be fully adequate for the reliable quantification of Pt and Pd. The data obtained in this investigation confirm that mushrooms are probative of a significant portion of the total exposure to PGEs due to the diet.

## 1. Introduction

Mushrooms are ubiquitous organisms that can be found in various environments, including both natural and human-impacted areas. They can remarkably adapt and thrive in several conditions [1,2]. In natural environments, like forests, mushrooms play essential roles as decomposers. They represent a group of living organisms characterized by significant nutritive, pharmaceutical, and ecological value [3]. The growth substrate is one of the factors that can highly affect the quality of edible mushrooms. Given the saprophytic characteristic, the mushrooms obtain their nutrients by absorbing the dissolved organic matter from the deadwood and other decay materials. Most of the elements can be bio-accumulated by the mushrooms, especially from the soil and substrates [4]. They can tolerate and grow in polluted urban and industrial areas. This adaptability is attributed to their unique biology and ecological characteristics. However, fungi are not limited to unpolluted environments. Several studies have shown that fungi, have a high ability to accumulate environmental contaminants, making them valuable in environmental monitoring and remediation projects [5,6,7]. Recent significant increasing interest in mushrooms, especially wild growing species, is associated with the accumulation of high levels of trace elements in their fruit bodies [3,8,9,10,11,12,13,14,15], which contain higher concentrations than in vegetables, fruits, and cultivated plants [16,17]. Mushrooms have the ability to accumulate various elements from substrate, which includes both the underlying soil or organic matter and the host trees they grow on. The process of metal and metalloid uptake and accumulation in mushrooms is complex and it depends on both species-specific (age of mycelium, morphological part of the fruiting body) and environmental (organic matter, pH, temperature, water content) factors [5,12].

Studies examining the chemical composition of wild species of mushrooms, including the analysis of Platinum Group Elements (PGEs), can provide valuable insights into ecological and environmental processes [18,19,20,21,22,23,24]. The presence and concentration of PGEs in wild species of mushrooms can vary depending on several factors, such as geographical location, environmental conditions, species-specific characteristics, and bioaccumulation processes. Automobile catalytic converters have been identified as a significant source of PGEs released into the environment.

Catalytic converters contain PGEs such as platinum and palladium, which act as catalysts to reduce harmful emissions from vehicles, mainly nitrogen oxides (NO_x_), carbon monoxide (CO), and hydrocarbons [25]. During the extraction and processing of noble metals, certain stages of the industrial processes can lead to the release of PGEs into the atmosphere. These emissions can occur through various pathways, such as stack emissions, fugitive emissions (unintentional releases), and waste disposal. The released PGEs can disperse and distribute in the environment [26,27,28,29].

Platinum and palladium are indeed elements that have gained attention, particularly due to their increasing presence in the environment. These are naturally occurring, and their levels have been amplified in the environment due to anthropogenic activities [30,31,32]. Emitted Pd and Pt are subsequently dispersed in the atmosphere or are subject to localized accumulation, for example, in road dust and adjacent roadside soil. Subsequently, they can be transported in air, road dust, soil, sewage sludge, and water, which eventually leads to bioaccumulation of these elements in living organisms via various pathways [33]. The uptake of platinum group elements is documented for certain plant species such as grass, cucumbers, maize, and mustard [31]. Therefore, it can be assumed that, analogously to heavy metals, some plant species possess the ability to accumulate noble metals [32]. The final chemical form of PGEs, their transformation in the environment, and their impact on living organisms are of primary importance. Several studies on the toxicity of platinum and palladium have demonstrated the potential adverse effects on human health [34,35,36].

The present study was undertaken to evaluate the accumulation of platinum and palladium in the fruiting bodies of *Boletus aereus*, since there is lack of data on baseline level of PGEs in mushrooms. Determining PGEs at environmentally and biologically relevant concentrations is a difficult task. Specific analytical methods are needed to measure the very low levels of PGEs in contaminated and uncontaminated materials to estimate background concentrations. Recent developments in the analytical sciences have enabled a better understanding of the environmental pathways of PGEs. In this study, we have set up a specific analytical protocol for determining Pd and Pt in mushroom samples by inductively coupled plasma mass spectrometry (ICP-MS).

The aim of the present study was: (1) determine the concentration of platinum and palladium accumulated in the fruit bodies for each sampling site; and (2) perform statistical analysis on the data obtained to assess the significance of platinum and palladium accumulation in the fruit bodies of *Boletus aereus*.

## 2. Material and Methods

### 2.1. Description of Sites and Sampling

This study was conducted to determine the content of Pt and Pd in fruiting bodies of *Boletus aereus* collected in Sicily (Italy). Sicily, the largest island in the Mediterranean, with an area total of about 25,000 km^2^, extends in latitude between 36° and 38° N and in longitude between 12° and 15° E. According to the Köppen macroclimatic classification, it can be defined as a temperate-humid climate region, i.e., the typical Mediterranean climate. The mean annual temperature varies from 11 °C to 20 °C, while the mean annual rainfall oscillates between 385 mm and 1192 mm [http://www.sias.regione.sicilia.it/pdf/Climatologia_PA.pdf (accessed on 6 August 2023)].

The complex geological history of Sicily is reflected in the great variety of rocks—sedimentary, magmatic, and metamorphic—that make it up.

**Figure 1:**
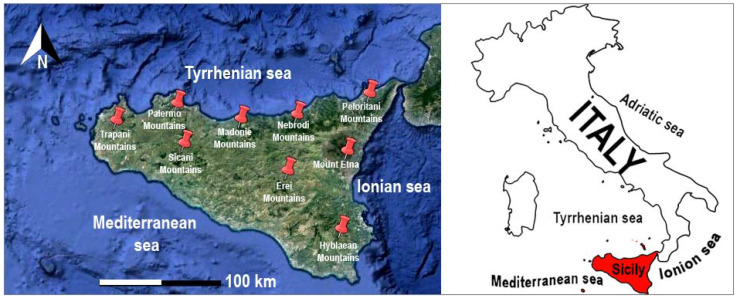
Sampling site location of mushroom *Boletus aereus* Bull.

The rocks of sedimentary origin are present in a large part of Sicily. They are ubiquitous in the northern part of the island (Monti di Trapani and Palermo, Madonie, and Nebrodi), in the central region (Monti Sicani), in the southern part (Erei mountains), and in the south-eastern part (Hyblaean mountains). The rocks of magmatic origin are in the eastern part of the island, corresponding with volcanic systems such as Etna, the largest active volcano in Europe. Finally, rocks of metamorphic origin are predominantly in the north-eastern part of Sicily in correspondence with the Peloritani Mountains. During the favorable season for fungal growth, *Boletus aereus* were collected at the selected study sites. Nine sampling areas were selected from several wooded areas of Sicily with different substrates (sedimentary and volcanic substrates) between the Trapani Mountains, Palermo Mountains, Sicani Mountains, Natural Regional Park of Madonie, Natural Park of Nebrodi, Peloritani Mountains, Erei Mountains, Hyblaean Mountains, and Natural Park of Mount Etna (Figure 1).

The sampling locations, mushroom species, and vegetation type are reported in Table 1.

*Boletus aereus* Bull. (Family: *Boletaceae*; Genus: *Boletus*) is a thermophilous fungal species, typically Mediterranean, growing and widely consumed in Italy, France, Greece, and Spain. It commonly grows in mycorrhizal association with various broad-leaved trees, especially *Quercus*, *Fagus*, *Castanea*, and sclerophyllous shrubs. Its presence decreases gradually toward northern Europe, and it is harvested from late spring to early autumn. *Boletus aereus* is appreciated for its pleasant fragrant aroma, taste, and nutritional value. These characteristics, along with its relatively easy availability, make *Boletus aereus* one of the most sought mushrooms for human consumption.

In Italy, picking mushrooms is regulated by regional and local laws, according to which the professional mushroom/fungi expert can collect a limited amount of mushrooms (Sicily Region, Law 02/, 2006, no. 4, art. 2 [37]). Mushroom samples were cleaned of debris using a brush, transported to the laboratory, and stored at −4 °C for up to 24h before sample preparation. Macroscopic descriptions of the fresh ascomata were noted, while the microscopic features were observed using an Olympus BX-51 (Tokio, Japan). For taxonomical identification, a series of monographs and keys were used by Breitenbach and Kränzlin [38], and Boccardo et al. [39].

The samples were cut using clean plastic knives and were dried (37 °C, overnight) in an electrically heated commercial dehydrator for mushrooms. After that, the dried fungal material was ground into powder using an agate mortar and then kept in polyethylene bags under dry conditions. Powdered mushrooms (~0.800 g) were digested with a mixture of 5 mL of 65% HNO_3_ (Suprapure Merck, Rahway, New Jersey) and 2.5 mL of 33% H_2_O_2_ (Suprapure Merck, Rahway, New Jersey). The digest was diluted to 40 mL using deionized water.

A volume of 5 mL of HNO_3_ (Ultrapure, Merck, Rahway, New Jersey) was added to approximately 800 mg of powdered mushrooms and digested for 24 h. Digestion was completed by adding 2 mL of H_2_O_2_ (Ultrapure, Merck) for another 24 h. The digestions were carried out at room temperature (T = 20 °C). After digestion, the solutions were diluted by adding 18 MΩ cm demineralized water to a volume of 40 mL and then filtered. Palladium and platinum determinations were carried out at the Department Scienze della Terra e del Mare (DiSTeM), University of Palermo, by inductively coupled plasma mass spectrometry (ICP-MS, Perkin-Elmer, Elan 6100 DRC-e, SD, CA, USA) after the addition of Y + Re as internal standards. All standard solutions were prepared with 18 MΩ cm demineralized water, the Pd and Pt CertiPUR standards (Merck, Rahway, New Jersey). The calibration standard addition technique was used for all element determinations to minimize matrix effects. Calibration curves ranging from 0.05 μg L^−1^ to 50 μg L^−1^ were constructed. For Pd and Pt determination, the ICP-MS was operated in DRC mode with CH_4_ as reaction gas to delete the polyatomic interferences. The operational limit of detection (LOD) for Pd and Pt was calculated as three times the standard deviation of the analyte concentration in blank samples (Pd: 0.009 μg L^−1^; Pt: 0.008 μg L^−1^). Certified reference material NIST SRM1515 Apple leaves was used for internal quality control. The reference material was spiked with Pd and Pt at two levels to provide 1 μg L^−1^ and 10 μg L^−1^. Spike recoveries were 86–98% for Pd and 93–99% for Pt at both concentrations.

### 2.2. Data Analysis

Data were analyzed statistically by the STATISTICA program (Tulsa, OK, USA), StatSoft version 6.0 (2001). All the tests in this study were considered significant at *p* < 0.05. The Shapiro–Wilk test was used to verify the normality of data distributions, and it showed that Pd and Pt were not normally distributed. The Mann–Whitney test, which makes no assumptions about the distributions and does not rely on distribution parameters, was used to verify any statistically significances differences between two or more independent groups.

### 2.3. BCF and DIR

The degree to which mushrooms had taken up elements from their substrate was expressed by the bioconcentration factor (*BCF*), obtained by dividing the element content in the fruiting body by the content of the same element in the corresponding soil on which it grew.

$$BCF=\frac{X_{mushroom}}{X_{substrate}}$$

*X* is the elemental concentration in mushrooms (ng g^−1^) and substrate (ng g^−1^).

It is a valuable indicator of the bioaccumulation potential of a substance or its exclusion.

The Daily Intake Rate (*DIR*) was estimated using the formula below:

$$DIR=\frac{C_{metal}\times C_{factor\;}\times D_{mushrooms\;intake}}{W}$$

where *C_metal_* is the elemental concentration in mushroom (ng g^−1^); *C_factor_* is a factor used to convert fresh mushroom weight to dry weight [40] and is set equal to 0.1; *D_mushroom_* is the daily intake of mushrooms, set at 300 g fresh mushrooms; *W* is the average adult body weight, considered to be 50 kg.

## 3. Results and Discussion

Palladium and platinum levels in analyzed samples are listed in Table 2. All concentrations were determined on a dry weight basis. The concentrations of investigated Pd and Pt in mushroom samples ranged from 0.31–3.09 ng g^−1^ for palladium and 0.21–4.22 ng g^−1^ for platinum.

**Figure 2:**
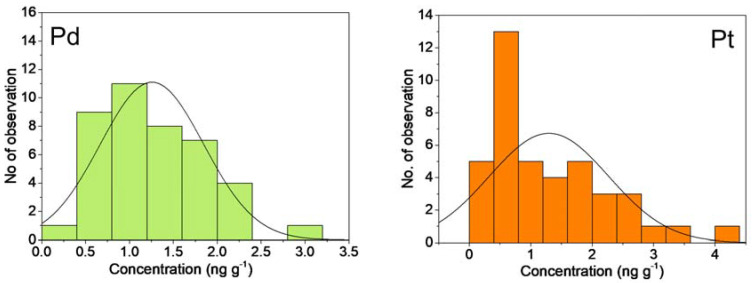
The frequency distribution of Pd and Pt in samples. Data are given in ng g^-1^.

The results of the Shapiro–Wilk test (W) (*p* < 0.05) show a non-normal distribution for Pd and Pt. To better characterize the data distribution, in Table 2, we reported the degree of asymmetry around its mode (Skewness coefficient) and the relative peakedness or flatness (Kurtosis coefficient). For both distributions, the Skewness value is near +1, which is considered a value that assesses the extent to which a variable’s distribution is positively asymmetrical. The Kurtosis values for Pd and Pt were 0.76 and 0.71, respectively, emphasizing a platykurtic distribution (Figure 2).

**Figure 3:**
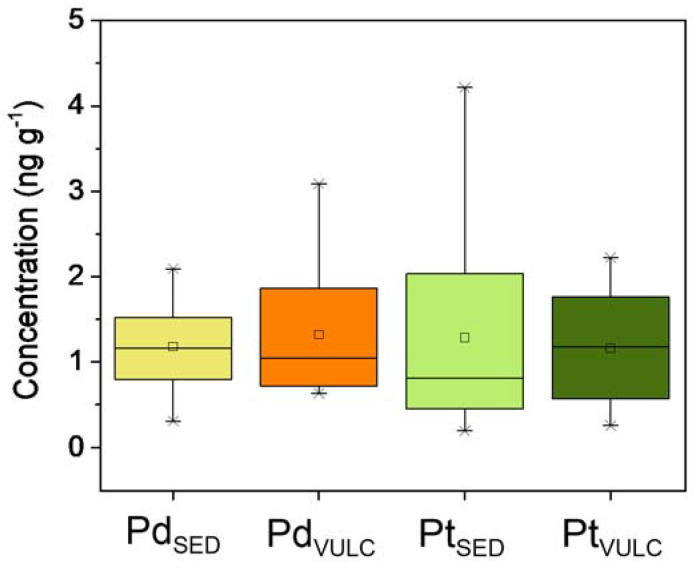
Box plots of Pd and Pt in the analyzed mushroom samples divided according to the sampling sites divided by main type of substrates of the study area (SED and VULC). Boxes delineate the interquartile range (25–75%) with the indication of the median (dark line); a small square inside the box marks the mean value; whiskers indicate the 10–90% range; points outside the box are minimum and maximum values. Data are given in ng g^−1^.

The coefficient of variation (CV%) calculated for *Boletus aereus* samples is 46 and 75% for Pd and Pt, respectively, highlighting a low variability mainly linked to the geology of the sampling sites and the distance from the road. The ratio Pt/Pd measured in samples varies between 0.13 and 4.84 with a median of 1.06, far from the typical values found in road dust samples which show a ratio between 7 and 12 [41]. Unfortunately, data on the PGE content in the air particulate matter within the study area are not available in the literature, making it difficult to explain the variability of the data. In Figure 3, we have reported the boxplot of Pd and Pt concentrations according to the sampling sites divided by main type of substrates of the study area (SED and VULC). From the figure, the palladium concentrations are similar in both environments; on the other hand, the concentrations of platinum appear to be different.

The non-parametric Mann–Whitney test (*p* < 0.05) suggests that the differences observed for Pt are not statistically significant, and the same is valid for palladium.

The contents of palladium and platinum determined in mushrooms are comparable or very low compared to the concentrations in various foods typically consumed by the population (Table 3) [12,40,41,42,43,44,45].

Although almost one-half of human exposure to PGEs has been estimated to occur through diet, only scant information on the likely levels of these elements in food is available in the literature [46,47].

Therefore, the palladium and platinum content determined in edible mushrooms is an important contribution to understanding how these metals can accumulate in different environmental matrices to evaluate a possible PGE risk for human health.

The increased number of cars and vehicles fitted with catalytic converters caused cars to release matter containing the above noble elements, which accumulate in the soil and plants or remain suspended in the air, being transported to large distances. Palladium and platinum can remain suspended in the air as aerosol particles for variable periods depending on particle size, wind speed, relative humidity, and precipitation; therefore, they can be transported thousands of kilometers [48]. Indeed, the concentration of these elements in the soil and plants has increased significantly during the last 10–15 years [49,50].

Djingova et al. [41] found low concentrations of platinum and palladium in a saprophytic mushroom collected from the vicinity of German highways. Platinum and palladium in mushrooms indicate that PGE particles from automotive exhaust converters or other sources have been dispersed and deposited in the surrounding environment [41]. There is poor information in the literature on the long-term effects of ingesting small amounts of PGEs and their bioavailability in the environment. According to some research, PGEs are mainly emitted in metallic form and exhibit low toxicity [51]. However, Pd and Pt can be converted into soluble forms, which become bioavailable and pose a serious threat to animals and plants [33,52]. Soil is one of the components of the environment most susceptible to PGE contamination [53,54]. Several studies have shown that human activity has caused an increase in the concentration of PGEs in the soil, especially near roads [55]. Pawlak et al. [51] compared the average concentration of Pt in different types of soil samples showing that the concentration of Pt in pristine soil is 0.14 μg kg^−1^; is 1.12 μg kg^−1^in agricultural soil; while it reaches 20.9 μg kg^−1^ in soil samples collected in areas close to roads. Furthermore, it has been observed that PGEs migrate in the soil, reducing in content with the soil depth [56]. The mobility of platinum and palladium in soil depends on many physicochemical factors, such as soil pH, redox potential, and salinity. The PGE transformation mechanisms that lead to their bioavailable forms are generally associated with various chemical reactions, including oxidation, complexation with organic ligands present in the soil, and biochemical reactions carried out by bacteria. The speciation of Pd and Pt as a function of pH indicated that these elements form hydroxyl complexes over the entire range of pH [57,58], in particular, Pt is predominant Pt(OH)_2_ at 1 < pH < 11.5, and Pd(OH)_2_ at 2 < pH < 12 [58,59]. The soil pH of the sampling sites varied from 4.24 to 6.66 [14] and it could be hypothesized that Pd and Pt are present in the form of hydroxides. A significant increase in PGE bioavailability in environmental samples can be observed in the presence of organic compounds [60]. The presence of humic acid in the soil leads to the immobilization of Pt salts in the form of almost soluble organic complexes. In contrast, other organic compounds increase the bioavailability of Pt and Pd [51].

The metal contents in mushrooms are commonly higher than those in agricultural plants, vegetables, and fruits. This suggests that fungi may have a very effective mechanism that allows them to absorb some metals and metalloids from the ecosystem [61,62,63,64,65,66].

The ability of mushrooms to concentrate or exclude specific metal ions was assessed by the bioconcentration factor (BCF) [67,68]. The BCF is defined as the ratio between the concentration of metal measured in the mushrooms and in the underlying soil where the mushrooms grew. The determined values of BCF indicate whether Pd and Pt are actively bioaccumulated (BCF > 1) or not (BCF < 1). The BCF calculated for *Boletus aereus*, considering the value of the continental crust [69] shows a BCF value > 1 (Table 4). The bioconcentration factor was also calculated using the concentrations of Pd and Pt determined on soil samples (Lo Medico F., personal communication) collected in some mushroom collection sites in the Sicily region. These BCFs show higher values than those calculated using the composition of the continental crust; therefore, inferring that the *Boletus aereus* is an accumulator of these metals from the substrate. There is no BCF data for Pd and Pt in the literature to compare the results obtained.

To at least partially understand the health risks deriving from the consumption of mushrooms, the daily intake rate (DIR) for Pd and Pt was determined. The DIR was calculated assuming the intake of a single portion of 300 g of fresh mushrooms (about 30 g of dried mushrooms), and their consumption by an adult weighing 50 kg. The calculations show that the worst-case scenario (maximum level detected) is 1.85 and 2.53 μg kg^−1^ of body weight/portion; on the other hand, the median value is 0.74 and 0.35 μg kg^−1^ of body weight/dose for Pd and Pt, respectively. To evaluate the potential health risks deriving from the intake of the two elements, in this study, we compared the results obtained with the permitted daily exposure (PDE) recommended by the guidelines of the European Medicines Agency [70] evaluated for Pd and Pt in 100 μg per day^−1^ (adult weighing 50 kg). The values found in this study are very low compared to the indicative values for the daily consumption of Pd and Pt. Repeating the same calculation and considering an average weight of an Italian adult equal to 70 kg, we obtain a median value of 0.53 and 0.26 μg kg^−1^ of body weight/dose for Pd and Pt, respectively. To our knowledge, the current literature does not report their effects at low environmental concentrations.

As known, the toxicity of an element is generally related to the type of compound (for example, metallic, inorganic, or organometallic salt), the route of exposure (for example, inhalation, dermal, oral), and the duration of exposure [71]. The available data evaluating the effects of exposure to PGEs continuously emitted into the environment and on potential toxicity are still limited today [34,35]. PGE concentrations in mushrooms are currently of no concern to people who consume these foods. However, it is noted that there is a possibility that palladium and platinum accumulation could become a problem in the future [72].

## 4. Conclusions

Limited information on platinum group element (PGE) accumulation in foods, including mushrooms, is available. Among the platinum group elements, palladium and platinum are used in various industrial applications, such as automotive catalysts, electronics, and jewelry. Although these occur naturally in the environment, their food concentrations are generally low. The few studies on Pd and Pt levels in fungi suggest that they do not accumulate significant amounts of these elements compared to other environmental matrices. However, it is worth noting that the levels of these elements in food products can vary depending on factors such as geographical location, soil composition, and cultivation methods. The results of the present study suggest that commonly consumed *Boletus aureus* mushrooms do not accumulate significant levels of Pd and Pt as demonstrated by BCF values, and their content is lower than in other food products. The low concentrations of Pd and Pt determined in edible mushrooms indicate that this type of food has a significantly low contribution to dietary exposure. This implies that the environmental impact and potential health risks associated with Pd and Pt through mushroom consumption are minimal.

It is important to note that these conclusions are based on the results of a specific study, and it is always helpful to consider a broader range of research when evaluating the health implications of consuming mushrooms or other food items.

The proposed methodological approach appears to be fully adequate for the reliable quantification of Pd and Pt. The data obtained in this investigation confirm that mushrooms are probative of a significant portion of the total exposure to PGEs due to the diet.

## Figures and Tables

**Table 1:** Sampling location and vegetation types.

No.	Sampling Location	Substrate	Vegetation Type
F1–F14	Nebrodi Mountains	SED	*Quercus cerris*, *Q. virgiliana*, *Q. suber*
F15–F16	Sicani Mountains	SED	*Q. sativa*, *Q.suber*
F17–F18	Peloritani Mountains	SED	*Q. virgiliana*, *Castanea sativa*, *Erica arborea*, *Genista e Prunus*
F19–F20	Madonie Mountains	SED	*Q. suber*
F21–F22	Palermo Mountains	SED	*Q. virgiliana*
F23–F24	Hyblaean Mountains	SED	*Q. virgiliana*, *Q. suber*, *Q. pubescens*, *C. avellana*, *C. sativa*
F25–F27	Erei Mountains	SED	*Eucalyptus camaldulensis*, *Q. virgiliana*, *Q. suber*
F28–F29	Trapani Mountains	SED	*Q. suber*
F30–F39	Mount Etna	VULC	*Q. virgiliana*, *Q. sativa*, *Q. suber*, *Fagus sylvatica*, *C. avellana*

**Table 2:** Basic statistical parameters of Pd and Ptin 39 mushroom *Boletus aereus*. Data are given in ng g^−1^.

	Min	Max	Mean	Std. Dev.	Median	P_10_	Q_25_	Q_75_	P_90_	Skewness	Kurtosis	CV%
Pd	0.31	3.09	1.26	0.59	1.18	0.63	0.81	1.71	2.04	0.79	0.76	46
Pt	0.21	4.22	1.30	0.97	1.08	0.38	0.54	1.87	2.52	1.07	0.71	75
Pt/Pd	0.13	4.84	1.35	1.15	1.06	0.20	0.33	2.07	2.73			

**Table 3:** Comparison of Pd and Pt elemental concentrations in several vegetable foods. Data are given in ng g^−1^; * Data are given in mg kg^−1^.

	Pd	Pt	Reference
*Boletus aereus* Bull.	0.52	0.53	this study
Mushrooms	0.03 *	0.01 *	Mleczek et al. [42]
Mushrooms	0.1 *	0.9 *	Mleczek et al. [12]
algae	6.6	0.56	Augustsson et al. [40]
berries, herbs	11	0.56	Djingova et al. [41]
Cerals	0.2	0.3	Kollander et al. [43]
Vegetable	69.6	0.024	Frazzoli et al. [44]
Potatos	0.5	<0.1	Ysart et al. [45]
Green vegetable	0.6	0.1	Ysart et al. [45]
Fresh fruit	0.4	<0.1	Ysart et al. [45]
Nuts	30	0.1	Ysart et al. [45]

**Table 4:** Bioconcentration factor in mushroom *Boletus aereus*.

ng g^−1^	Pd	Pt	BCF Pd	BCF Pt
Upper continental crust	0.52	0.5	2.4	2.5
Sicilian soil	0.21	0.2	5.9	6.2

## Data Availability

The data presented in this study are available on request from the corresponding author.
